# Brain Network Activation Analysis Utilizing Spatiotemporal Features for Event Related Potentials Classification

**DOI:** 10.3389/fncom.2016.00137

**Published:** 2016-12-20

**Authors:** Yaki Stern, Amit Reches, Amir B. Geva

**Affiliations:** ^1^ElmindA Ltd.Herzliya, Israel; ^2^Electrical and Computer Engineering, Ben-Gurion University of the NegevBeersheba, Israel

**Keywords:** BNA, ERP, EEG, STEP, functional connectivity, machine learning

## Abstract

The purpose of this study was to introduce an improved tool for automated classification of event-related potentials (ERPs) using spatiotemporally parcellated events incorporated into a functional brain network activation (BNA) analysis. The auditory oddball ERP paradigm was selected to demonstrate and evaluate the improved tool.

**Methods:** The ERPs of each subject were decomposed into major dynamic spatiotemporal events. Then, a set of spatiotemporal events representing the group was generated by aligning and clustering the spatiotemporal events of all individual subjects. The temporal relationship between the common group events generated a network, which is the spatiotemporal reference BNA model. Scores were derived by comparing each subject's spatiotemporal events to the reference BNA model and were then entered into a support vector machine classifier to classify subjects into relevant subgroups. The reliability of the BNA scores (test-retest repeatability using intraclass correlation) and their utility as a classification tool were examined in the context of Target-Novel classification.

**Results:** BNA intraclass correlation values of repeatability ranged between 0.51 and 0.82 for the known ERP components N100, P200, and P300. Classification accuracy was high when the trained data were validated on the same subjects for different visits (AUCs 0.93 and 0.95). The classification accuracy remained high for a test group recorded at a different clinical center with a different recording system (AUCs 0.81, 0.85 for 2 visits).

**Conclusion:** The improved spatiotemporal BNA analysis demonstrates high classification accuracy. The BNA analysis method holds promise as a tool for diagnosis, follow-up and drug development associated with different neurological conditions.

## Introduction

The high dimensional and complex nature of electroencephalogram (EEG) recordings is a result of the spatiotemporal structure of the neurophysiological signals. Traditional methods for EEG and event-related potential (ERP) analysis follow waveform morphology over time at selected electrode locations, using either time-domain or frequency-domain tools, while neglecting the spatiotemporal dynamics associated with the electric fields at the scalp (Brunet et al., 2011; Michel and Murray, 2012).

The entire spatiotemporal space involves a large quantity of data, not all of which are clinically significant, and necessitates a reduction in dimensions. However, existing data reduction methods sometimes rely on a restriction of the solution space either in the temporal or spatial dimension and as a consequence, a large quantity of the spatiotemporal dynamics may be lost (Hasson-Meir et al., 2011). Therefore, it is more desirable to utilize data-driven analysis methods where the data guides the choice of the model while minimizing a priori assumptions (Hasson-Meir et al., 2011). One known method of data-driven analysis is microstate analysis (Lehmann and Skrandies, 1980; Lehmann, 1987). This analysis assumes that a task-related brain activation can be segmented into specific functional states known as microstates, which are stable for about 80–120 ms, and which are each represented by the entire recording space at a specific time point (topographic map). The only temporally dynamic component in the microstate model is the transition between states. Thus, each state lacks the dynamic spatiotemporal evolution of the electric field at the scalp (Dimitriadis et al., 2013; Zoltowski et al., 2014; Khanna et al., 2015; Mheich et al., 2015).

In the work presented here, an improved spatiotemporal brain network activation (BNA) analysis for automated pattern classification of ERP data is presented. Here, “events” are defined as spatiotemporal ERP extreme points (“peaks”) and their surrounding activity. Cortical activity is segmented into discrete parcels, each containing spatial, temporal, and frequency domain information surrounding ERP peaks. Thus, rather than extracting stationary topographic maps that are derived from the entire recording space, as in microstate analysis, BNA analysis with spatiotemporal parcellation captures spatiotemporal dynamics embedded within each parcel. Additionally, spatiotemporal segmentation allows separate events, spatiotemporal parcels (henceforth “STEPs”), to be warped without distorting the entire signal, which is not possible with methods such as Woody (1967), mean shift (Comaniciu and Meer, 2002) and other well-known warping methods (Bellman and Kalaba, 1959; Efrat et al., 2007). This improved warping method enables a better alignment among the events of individual subjects and, thus, a better group representation. The improvement in the current BNA analysis is related to the neurophysiological events on which the network is based. While in previous BNA analysis (Shahaf et al., 2012; Reches et al., 2013; Kontos et al., 2016) the events were temporal peaks at a single electrode, here BNA analysis relies on a spatiotemporal parcellation method (Stern et al., 2012).

In order to evaluate the BNA algorithm using non-simulated data, the oddball ERP paradigm was selected. Three aspects of the oddball task make the paradigm a suitable choice for this evaluation. First, the oddball ERP paradigm is a well-known paradigm in cognitive neuroscience and its waveform and components are extensively explored (Key et al., 2005). Second, the 3-stimulus oddball paradigm used here contains a standard stimulus (the “Frequent” stimulus), which is presented frequently, and two non-standard stimuli (the “Target” and “Novel” stimuli), which are presented rarely. The Target and Novel stimuli are thought to share partially common neuronal activation networks (Ebmeier et al., 1995; Kirino et al., 2000), leading to similar waveform manifestations, which in turn make the differentiation between those two stimuli challenging (Linden, 2005; Polich, 2007). Finally, alterations in the oddball ERP components have been reported in a variety of neurological conditions such as mood disorders, schizophrenia, dementia, and traumatic brain injury (Duncan et al., 2009). Thus, the ability to correctly classify minute and consistent changes in the ERP waveform would suggest that the BNA algorithm may have merit as a tool for diagnosis, follow-up and drug development associated with different neurological conditions.

**Figure 1:**
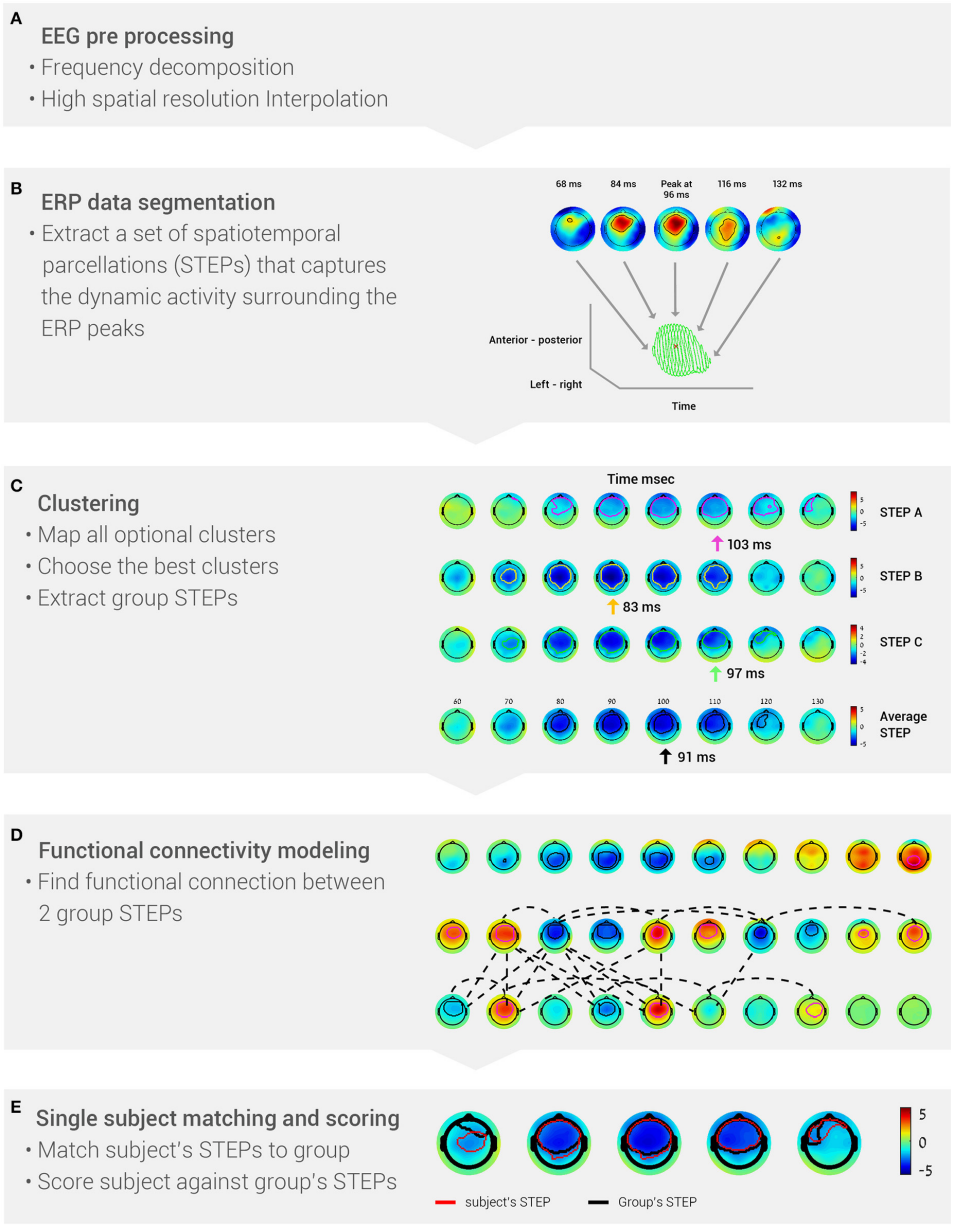
**The five stages of the BNA algorithm analysis. (A)** EEG data are pre-processed into frequency bands and a high-resolution grid. **(B)** Event-related potentials are segmented to identify the peaks (STEPs). **(C)** Data are clustered to extract group STEPs. **(D)** Functional networks are determined from the group STEPs. **(E)** Score each subject's BNA analysis against the group data.

The BNA algorithm comprises the following stages (Figure 1): (A) EEG pre-processing; (B) ERP data segmentation, i.e., segmenting each subject's data into STEPs; (C) Clustering, which generates a set of STEPs that are common to the majority of the group members; (D) Functional connectivity estimation between two group STEPs, which is determined from the temporal synchronization between them; and (E) Single-subject matching and scoring. At this stage, the degree of similarity between a single subject and the group is calculated.

The purpose of this study was to present the spatiotemporal BNA algorithm. In order to evaluate the algorithm, we examined: (1) the reliability of BNA analysis by assessing test-retest repeatability and (2) the utility of BNA analysis as a classification tool. For that purpose, BNA analysis was employed to discriminate between ERPs elicited in response to Target and Novel stimuli of an “oddball” paradigm, using an SVM classifier (Lotte et al., 2007). The premise was that these findings might extend to the discrimination of brain responses between healthy controls and clinical populations.

## Materials and methods

### Data acquisition and experiment

#### Subjects

Two groups of healthy, right-handed male and female subjects from four different clinical research centers participated in the study. The first group (“Group A” from Lowenstein Hospital, Ra'anana, Israel) included 40 subjects (23 females) whose ages ranged from 23 to 64 years. The second group (“Group B”) included 70 subjects (26 females) whose ages ranged from 14 to 25 years. Participants in Group B were recruited from Clinilabs Clinical Research Organization, New York (Clinilabs, *N* = 15), University of Pittsburgh Medical Center (UPMC, *N* = 15) and University of Michigan Neurosport (*N* = 40). All participants signed informed consent documents to participate in the study, which was approved by the Ethics Committees of the respective centers.

#### Task and data

All subjects performed an auditory oddball task. For each subject, there were 600 trials, of which 80% were 2000 Hz stimuli (Frequent), 10% were 1000 Hz rare stimuli requiring a response (Target) and 10% were rare non-targets that consisted of various sounds (Novel). Stimuli were separated by 1500 ms intervals. Subjects were told to fix their gaze on a sign in the middle of a screen. Stimuli were delivered using a headset and the output level was 70 dB SPL.

Subjects in Group A participated in three sessions (visit 1, visit 2, and visit 3, hereafter, V1, V2, and V3, respectively) that were spaced 1 week apart. EEG recordings were obtained using a 64-channel Active Two system (Biosemi, Amsterdam, Netherlands) with a sampling rate of 256 Hz. Group B participated in two repeated sessions (V1 and V2) and recordings were obtained using a HydroCel Geodesic Sensor Net of 128 channels and a Net Amps 300 amplifier (Electrical Geodesic Inc., Eugene, Oregon) with a sampling rate of 250 Hz. Artifact removal included noisy electrode removal (extensive temporal sections of the signal with an amplitude outside the range of ±100 μV or high dissimilarity to neighboring electrodes), noisy epoch removal (epochs with amplitudes outside the range of ±100 μV or amplitudes that were more than 7 standard deviations from the mean) and eye artifact correction using independent component analysis (ICA). All artifact removal stages were done using EEGLAB software (v. 9.0.4s, Delorme and Makeig, 2004).

### BNA algorithm

The aim of the improved BNA algorithm is to parcel the EEG activity into major spatiotemporal events surrounding the ERP peaks in order to generate brain networks at the group level consisting of functional connections between these events. To achieve this aim, the BNA algorithm comprises the following stages: pre-processing, EEG data segmentation, clustering, functional connectivity estimation, and single subject matching and scoring (calculated against group characteristics). The five basic stages of the BNA algorithm analysis are summarized in Figure 1. Each of these stages will now be described in more detail. In the previous version of the BNA algorithm (Shahaf et al., 2012; Reches et al., 2013), an event was defined as a temporal peak at a single electrode (i.e., without temporal or spatial range), while in this paper an event was defined as a STEP (i.e., one event represented activity at few neighboring electrodes within a certain time frame around the peak; Stern et al., 2012).

#### Pre-processing

For each subject, ERPs were first decomposed into four conventional frequency bands (Figure 1A), δ (0.5–4 Hz), θ (3–8 Hz), α (7–13 Hz) and β (12–30 Hz). Digital filtering was accomplished with a linear-phase FIR filter design using least-squares error minimization and reverse digital filtering. Next, a high-resolution spatial grid of brain activity was calculated (Figure 1A). This stage resulted in a 3-dimensional matrix, with 2 dimensions for spatial locations of the activity on the head (left-right and anterior-posterior) and one temporal dimension. The spatial dimensions were estimated by projecting the 3D electrode array locations into 2D space. For each time sample, the recorded activity was interpolated into a higher resolution 2D grid by use of cubic spline interpolation (the grid size was 33 × 37 pixels, which reflects a resolution that is 4 times greater than a 10–20 electrode placement system).

#### High-resolution ERP data segmentation

After pre-processing, the high-resolution ERP activity of each subject in each of the four frequency bands was segmented into: (1) spatiotemporal ERP peaks and (2) their associated surroundings. This segmentation resulted in a set of spatiotemporal parceled events (STEPs), i.e., a set of segments that encapsulate the dynamic spatiotemporal information surrounding the ERP peaks (Figure 1B). A spatiotemporal ERP peak was defined as a local extremum of the amplitude in time and space. Each peak could therefore be described with basic attributes: amplitude, time, and spatial location (left-right; posterior-anterior). The peak's surroundings were defined as the ERP activity (amplitude) around the peak in the temporal and spatial domain. The threshold for the surroundings was defined as half the absolute value of the peak's amplitude. The goal of the segmentation stage was to reduce the subject's entire brain activity into a set of STEPs.

#### Clustering

The BNA analysis method uses a graph representation (vertices and edges) to depict the group-level brain activity as a network evolving in time, location and frequency (for a review regarding network models of cognitive processing see Sporns, 2014). In this network, the group STEPs are the vertices connected by edges that represent the functional connections showing temporal synchronization. Network generation is a two-stage process. First, the group STEPs are extracted by clustering the STEPs of each subject included in the group. Second, a functional connectivity model was generated (the next stage in BNA analysis, see below).

The goal of clustering was to discover a set of group STEPs that represented a spatiotemporal event common to at least 70% of the subjects. To extract the group STEPs, a clustering procedure was performed at a given frequency band. The input data for the clustering procedure were composed of all the STEPs extracted at the single-subject level. A STEP at the group level has the same characteristics as a STEP at the single subject level, i.e., a peak and surrounding activity. The clustering procedure comprised three stages: (a) mapping all optional clusters under both spatial and temporal constraints; (b) choosing the best clusters using a greedy algorithm; and (c) generating a group STEP for each cluster selected in the previous stage (Figure 1C). For further details regarding the clustering procedure, see Supplementary Materials.

#### Functional connectivity modeling

At this stage, the functional connectivity between two group STEPs was determined. The connectivity between two group STEPs was defined as a temporal synchronization between them. Two synchronization measures were calculated: spatiotemporal peak synchronization and rise-time synchronization. The spatiotemporal peak synchronization was defined as the temporal difference (Δt) between the spatiotemporal peaks of two connected STEPs. The rise-time onset of a STEP is the earliest point in time in which an activity in the STEP begins to occur. The STEP rise-time synchronization was defined as the difference in rise time onset (Δt) between two connected STEPs. A pair of STEPs was defined as connected if Δt between those STEPs was less than or equal to 30 ms. The temporal constraint on the connectivity between STEPs needed to apply to at least 70% of the subjects whose data served as input for clustering. The resultant set of events and connections characterized the group-common brain activity (Figure 1D).

#### Single subject matching and scoring

The goal of this stage was to calculate single subject features against a set of group STEPs and connections. As mentioned above, a single subject representation is similar to that of the group in terms of peaks and surroundings. However, since a group has less spatiotemporal events than a single subject due to the clustering procedure, it was necessary to match each STEP at the group level to its corresponding STEP at the single subject level (Figure 1E). After the matching process was completed, single subject features could be calculated (Figure 1E). Two types of features were generated for each STEP: one is a topographic similarity score and the other is the average global field power (GFP), which is an attribute of the individual subject's STEP. The topographic similarity score reflected the similarity to the group's STEP dynamics in terms of the timing of the activity, spatial location on the head, and the evolution of the activity over time (Equations 1–3). GFP was the energy of the activity that was measured (Equation 4), which reflects changes in electric field strength (Lehmann and Skrandies, 1980). The topographic similarity and GFP are analogous to the conventional ERP peak latency and amplitude measures, respectively. In addition, the two connectivity features that are based on temporal synchronization were also extracted. The output of the single subject matching and scoring stage was a set of features corresponding to a STEP or connection in the network of the group.

(1) $$\begin{array}{l} STcov\left(ST^{s},ST^{g}\right)=\\ \overset{all\;voxels}{\underset{x,y,t}{\sum }}\left(S{T^{s}}_{x,y,t}-\;\hat{ST^{s}}\right)\;*\;\left(S{T^{g}}_{x,y,t}-\;\hat{ST^{g}}\right) \end{array}$$

*ST*^*s*^, *ST*^*g*^ are the STEPs of the single subject and the group, respectively. A STEP is represented by a 3-dimensional matrix, where voxels of the STEP contain the amplitude values and Nan values are assigned outside the STEP range. *x, y, t* are the STEP voxel indices, where *x* represents the anterior-posterior axis, *y* represents the left-right axis, and *t* represents the temporal axis. $\hat{ST^{s}},\hat{ST^{g}}$ are the amplitude averages of the STEP

(2) $$\begin{array}{l} STvar\left(ST\right)=STcov\left(ST,ST\right) \end{array}$$

(3) $$\begin{array}{l} TopoCorrCoeff\left(ST^{s},ST^{g}\right)=\frac{STcov\left(ST^{s},ST^{g}\right)}{\sqrt{STvar\left(ST^{s}\right)\;*\;STvar\left(ST^{g}\right)}} \end{array}$$

*ST*^*s*^, *ST*^*g*^ are the STEPs of the single subject and the group, respectively. If *ST*^*s*^ doesn't fulfill the spatiotemporal windows constraints with respect *ST*^*g*^, *TopoCorrCoeff* (*ST*^*s*^, *ST*^*g*^) = 0

(4) $$\begin{array}{l} GFP=\;\sqrt{\frac{1}{N}\overset{N}{\underset{i=1}{\sum }}amplitud{e_{i}}^{2}} \end{array}$$

Where N is the STEP size (number of voxels) and *i* is a single index representation of one *x, y, t* combination. Only combinations of *x, y, t* indices that are in the STEP boundaries are assigned to *i*.

### Classification

All of the features that were described in “Single subject matching and scoring” above were scaled and entered into a classifier. This set of features was calculated against the Target and Novel networks generated for the data of Group A at V3 (see the “Subjects” sub-section in “Data acquisition and experiment” above). A linear support vector machine (SVM) classifier was used to classify responses to the Target stimulus from responses to the Novel stimulus. SVM is a commonly used classifier for multiple feature classification (Lotte et al., 2007). In this study, the linear SVM classifier was performed using scikit-learn python toolbox (v. 0.14a1, Pedregosa et al., 2011). In order to avoid overfitting, the C parameter was set to *C* = log(–3.5) (Fan et al., 2005). The distance of the subject from the separating plane determined the class of the subject, and the set of coefficients that generate the separating plane determined the strength of each feature in the classification. In order for the coefficients to adequately represent the strength of each feature, the features have to be on a common scale and, therefore, they were scaled before being entered into the classifier (Equation 5).

(5) $$\begin{array}{l} \tilde{{\text{x}}_{i}}=\frac{x_{i}-\hat{x}}{\sigma_{x}} \end{array}$$

Where *x*_*i*_ is a variable of a specific feature, $\hat{x}$ is the average of all variables of the specific feature and σ_*x*_ is the standard deviation of the specific feature.

### Experimental design

The oddball ERP task was selected in order to evaluate the BNA algorithm. The study design relied on two main principles. First, the Novel and Target stimuli are two different conditions that share partially common neuronal activation networks (Ebmeier et al., 1995; Kirino et al., 2000), leading to similar waveform manifestations. Therefore, those conditions can be viewed as analogous to two different clinical conditions, e.g., healthy controls and a patient group with a disorder in common. Thus, we tested the ability of the BNA algorithm with the SVM classifier to correctly classify the two types of responses (detailed reviews on Target-Novel discrimination can be found in Polich, 2007 and Linden, 2005). Second, to evaluate the generalizability of the method, the classifier was trained on Group A at V3, and applied to a validation set that included Group A and a test set that included Group B, V1 and V2 in each. Group A and B differed in age range and in terms of the technical details related to the data collection, such as the recording system, the number and placement of recording electrodes, and the sampling rate. These differences may attest to the generalizability of the method and of the findings presented here. The classifier's performance was evaluated using a receiver operating characteristic (ROC) curve and the stability of the known ERP components was tested using intra-class correlation (ICC) (Equation 6).

(6) $$\begin{array}{l} ICC=\frac{MS_{b}-MS_{w}}{MS_{b}+\left(k-1\right)*\;MS_{w}} \end{array}$$

*MS*_*b*_, *MS*_*w*_ are the ANOVA's mean squares between and within calculations, where each subject was considered as a group, and *k* is the number of visits.

## Results

### Reference BNA model at visit 3

**Figure 2:**
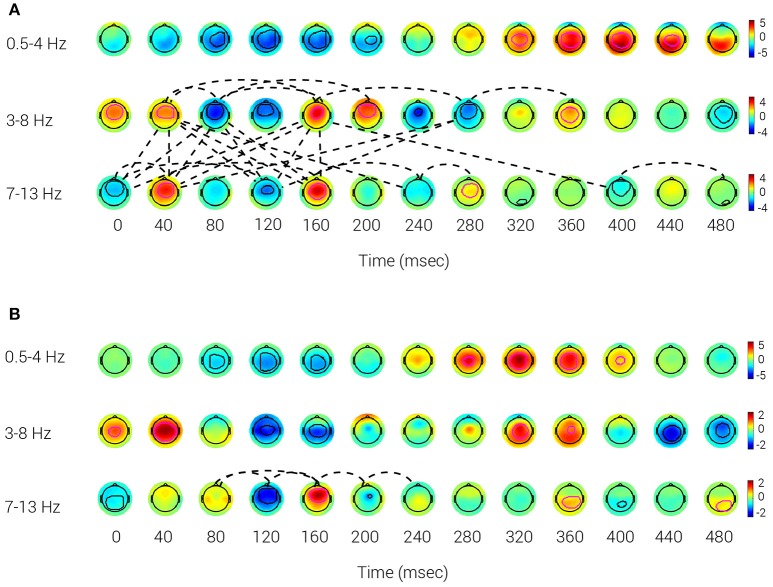
**Reference BNA model**. The activity patterns of the network derived from Target **(A)** and Novel **(B)** stimuli of Group A. The contours (thick lines) that appear inside the potential maps circumscribe each STEP's peak and surroundings. A magenta contour represents a positive polarity group STEP, whereas a black contour represents a negative polarity group STEP. The dotted lines are the connections between the group STEPs. The data were collected at V3. **(A)** Target: Positive polarity group STEPs, corresponding to the known P300 ERP component at 320–440 ms, can be observed in the δ band (0.5–4 Hz). Target N100 is the negative polarity STEP with a spatiotemporal peak at ~100 ms and P200 is the positive polarity STEP with a spatiotemporal peak at ~180 ms, both occurring in the θ band (3–8 Hz). **(B)** Novel: A P300 component (at 280–400 ms) can be observed in the δ frequency band. N100 is the negative polarity STEP with a spatiotemporal peak occurring at ~145 ms in the θ frequency band, while no STEP corresponding to P200 emerged.

The reference BNA model for the Target and Novel stimuli data collected from Group A at V3 was calculated to obtain a group representation of the activity and connectivity for those stimuli conditions (Figures 2A,B). The evolution of the STEPs over time for each stimulus condition is displayed for three frequency bands: δ (0.5–4 Hz; top row), θ (3–8 Hz; middle row) and α (7–13 Hz; bottom row), respectively. Looking at the STEPs associated with the Target and Novel stimuli, the STEPs corresponding to the P300 ERP component (Target: 320–440 ms; Novel: 280–400 ms) were observed in the delta frequency band. The Target N100 (~100 ms) and The P200 (~180 ms) are both occurring in the theta frequency band (Key et al., 2005). For the Novel stimulus, Novel N100 (~145 ms) is occurring in the theta frequency band, while no STEP corresponding to the Novel P200 emerged. The known P300 ERP component was elicited by Target and Novel stimuli, with spatiotemporal peaks occurring at approximately 375 ms (Target) (Figure 2A) and 335 ms (Novel) (Figure 2B) following stimulus onset. The scalp distribution of the P300 peak was more posterior in the Target condition (320–440 ms) compared to the more central peak distribution in the Novel condition (280–400 ms). Taken together, the Novel STEP appeared earlier and was more centrally located than the Target STEP, which had a more posterior distribution, similar to other reports in the literature regarding the P3a and P3b components (Polich, 2007).

Regarding functional connectivity, the BNA analysis of the Target condition showed denser connectivity than that of the Novel condition BNA analysis. The Target network connectivity was prominent over the 40–240 ms interval and implicated connections between STEPs within the theta and alpha bands as well as cross-frequency connections between theta and alpha STEPs (Figure 2A). A similar connectivity pattern also occurred for the Target network at V1 and V2 (see Supplementary Material, Figures S1A,B) although the densest connectivity pattern appeared at V3 (Figure 2A). This similar connectivity pattern that emerged across multiple visits (V1 through V3) indicated that features extracted by BNA were repeatable over time. In contrast to the Target condition, connections in the Novel stimulus condition were scarce and appeared only within the alpha frequency band during the 80–240 ms interval at V3 (Figure 2B). The relative scarcity of cross-frequency connections between theta and alpha STEPs in the Novel condition could also be observed at V1 and V2 in the 80–160 ms interval (see Supplementary Materials, Figures S2A,B). In addition, no within-theta connections were evident in the Novel condition in any of the three visits.

### Single-feature repeatability

Table 1 displays the ICC values for the topography and GFP scores of the N100, P200, and P300 components. Interestingly, the ICCs of the GFP score were higher than those of the topography score, indicating that the steepness of the STEP gradient may be more repeatable in some cases than the overall structural similarity between STEPs (i.e., the evolution of brain activation over time - see Material and Methods). To show the capabilities of conventional ERP analysis, the ICC values of conventional ERP measures (i.e., latency and amplitude) were also reported (Table 1). In general, values of ICC below 0.4 are considered “poor,” values between 0.4 and 0.75 are considered “fair” to “good” and values above 0.75 are considered “excellent” (Fleiss, 1986). As can be observed, the ICC values associated with the amplitude of the classic ERP components were comparable or just slightly higher than those exhibited by the GFP scores. For BNA analysis, the ICC values of the GFP scores were good (around 0.75) while the values of the topography scores ranged between fair to good. However, the ICC values associated with the latency of the ERP components were poor to fair and ranged from 0.17 to 0.46 (Table 1).

**Table 1:** **Repeatability of known ERP components**.

		BNA analysis^*^		**Standard ERP analysis**	
		**Topo**	**GFP**	**Latency**	**Amplitude**
N100	Target	0.51	0.77	0.17	0.81
	Novel	0.72	0.75	0.41	0.75
P300	Target	0.77	0.72	0.46	0.82
	Novel	0.72	0.82	0.31	0.85
Target—P200		0.56	0.68	–	–

* *ICC was calculated using Group A V1 and V2*.

### SVM classifier

**Figure 3:**
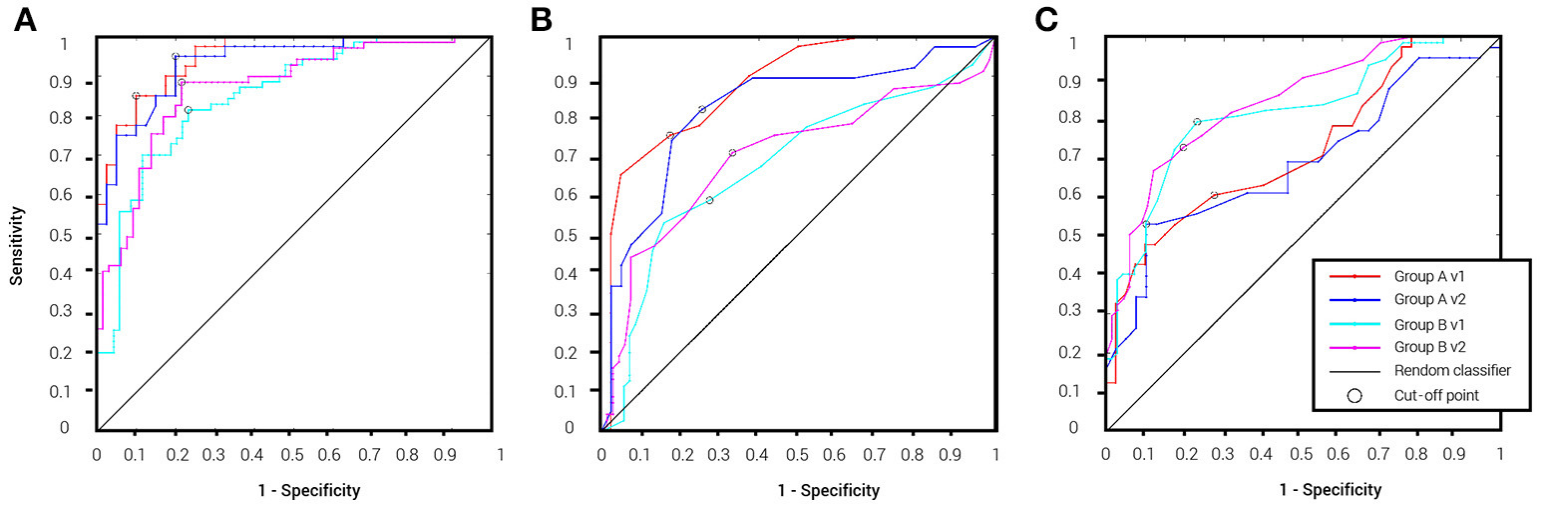
**ROC curves for the four group-visit combinations (Group A and B, V1 and V2)**. **(A)** ROC curves for BNA analysis. **(B)** ROC curves for the ERP N100 latency. **(C)** ROC curves for ERP P300 latency. The cut-off point determines the sensitivity and specificity of the classification and its utility as a clinical tool.

Table 2 details the separation ability scores (AUC values) obtained by BNA analysis for each group and visit. As with the ICC, the Target-Novel separation ability was also reported for conventional ERP peak identification analysis (Table 2). In general, AUC values between 0.5 and 0.7 are considered “poor” separation, values between 0.7 and 0.9 are considered “moderate” separation, and values between 0.9 and 1 indicate “high” separation (Greiner et al., 2000). As can be observed, the BNA AUC values for the validation group (Group A) were high (0.93 and 0.95 for V1 and V2, respectively) whereas for the test group (Group B) the AUC values were in the upper range of the moderate separation (0.81 and 0.85 for V1 and V2, respectively). The AUC values of the ERP component measures (latency and amplitude of N100 and P300) ranged only between poor to moderate for each combination of group and visit (the rows in Table 2). ROC curves for the four group-visit combinations (Group A and B, V1 and V2) were plotted for BNA and for the ERP measures with the highest accuracy, i.e., N100 latency and P300 latency (Table 2 and Figures 3A–C, respectively). Note that only for the P300 latency (Figure 3C) did the separation ability of Group B outperform that of Group A.

**Table 2:** **The separation ability (AUC values) of BNA analysis and standard ERP analysis**.

		**BNA + SVM**	**Standard ERP analysis**			
			**N100 Latency**	**N100 Amplitude**	**P300 Latency**	**P300 Amplitude**
Group A	V1	0.93	0.88	0.58	0.72	0.71
	V2	0.95	0.81	0.63	0.68	0.71
Group B	V1	0.81	0.68	0.65	0.81	0.65
	V2	0.85	0.70	0.62	0.84	0.65

## Discussion

In this study, we have presented a methodology, termed BNA analysis, that can extract information about functional brain networks from ERP activity, consisting of major events that encapsulate the dynamic spatiotemporal information embedded within the ERPs and their temporal relationships. This methodology is based on previous work on spatiotemporal representation (Stern et al., 2012) and functional connectivity of ERPs (Shahaf et al., 2012; Reches et al., 2013; Kontos et al., 2016). BNA analysis was evaluated on ERPs from Target and Novel stimuli (elicited in the context of an “oddball” paradigm). Using supervised training (SVM), the method allowed users to choose a subset of network features that were generated in an unsupervised way that demonstrated both high stability and separation ability. The novelty of the present study lies in the notion that each neurophysiological event generated by distributed neuronal populations and brain regions has a unique spatiotemporal structure and there are dynamic functional relationships among those events that can be represented by a network (Sporns, 2014). In addition, because the dynamic functional connectivity, as depicted by BNA analysis, contains spatiotemporal information at a millisecond resolution, BNA analysis allows the depiction of the temporal dynamics of functional connectivity.

The separation and repeatability of Target and Novel conditions in the context of an ERP oddball paradigm were used to demonstrate the clinical advantages of BNA analysis. As the results indicate, the repeatability and separating ability of BNA were high (Tables 1, 2, respectively). Moreover, although the ICC values of the single features ranged between fair to good (Table 1), the AUC values (Table 2) were either good or excellent, indicating very good separation ability obtained by BNA analysis. It is noteworthy that training was performed on the data of V3 (Group A) whereas the ICC and AUC analyses were performed on the data of V1 and V2 (separately for Group A and B). Therefore, the high AUC values obtained for Group A or B relative to the AUC values obtained for the standard ERPs cannot result from overfitting. While the AUC results for Group A (the validation group) are not overfitted in terms of using the same data, there may be some tendency to overfitting by using the same subjects recorded in different visits.

From the AUC values of the standard ERPs (Table 2), we noticed that, in the majority of cases, higher AUCs were obtained with the latency than the amplitude measure. The ICCs associated with the ERP latency were poor (Table 1). The dissociation of the AUC and ICC values is related to the calculation of those two measures. A high AUC value reflects a narrow score range for each of the clinical cases that need to be separated, while the ICC calculation is a more complex equation and is based on the relationship between the group variance and the single-subject variance (Equation 6). A high ICC value can be caused by high group variance or low single-subject variance. If the high ICC value results from high group variance, a high AUC value is unlikely to be achieved. A combination of high AUC and high ICC values reflects a score that is repeatable with low group and single-subject variances, which is a desirable situation for clinical use. The dissociation between the ICC and AUC values that was demonstrated by the standard ERP analysis (Tables 1, 2, respectively) underscores the advantage of using BNA analysis over conventional ERP analysis techniques. A clinical classification tool should demonstrate both high stability and separation ability, criteria that are not jointly met by the ERPs in this case.

The STEPs generated in the reference BNA model represent the major ERP components that are part of the sensory and cognitive process of the stimuli, as reported in the oddball task literature (Key et al., 2005; Duncan et al., 2009). The connections between those components, N100, P100, P200, and N300 (the N200 component occurring at ~260 ms) are also associated with neurophysiological processes, such as stimulus processing mechanisms (the P100-P200 complex, Hackley et al., 1990; Luck and Hillyard, 1994; Johnstone et al., 1996; Tonnquist-Uhlén, 1996; Dunn et al., 1998), automatic perception of auditory objects (the N100-P200 and the P200-N300 complexes, Mueller et al., 2008; Bien et al., 2012), as well as selective attention and stimulus evaluation processes (the N300 and P300 complex, Patel and Azzam, 2005; Lee et al., 2011). The connectivity between the theta and alpha frequency bands that was prominent in the target condition is similar to previous findings that emphasize the role of pre-stimulus alpha activity in modulating auditory ERPs and the cross-spectral interaction between resting alpha and delta/theta band activation associated with ERP components (Lee et al., 2011).

The low number of connections associated with the Novel condition might be associated with the known habituation of the response to novelty across trials (Debener et al., 2002; Yamaguchi et al., 2004; Murty et al., 2013) or with reduced connectivity as a function of repetition (Garrido et al., 2009). An alternative plausible explanation is that individual differences in the rate of habituation might have increased the inter-subject variability in functional connectivity (Mueller et al., 2013). If this was indeed the case, then this increased variability might have hampered the ability of BNA analysis to identify common connectivity patterns across individual subjects in the Novel condition, thus explaining the scarcity of connections between the nodes in the Novel network. The connectivity between two STEPs was defined as the temporal synchronization between them. With the developing field of imaging connectomics (Fornito and Bullmore, 2015) and its relation to cognitive processes (Sporns, 2014), other modeling methods of functional interactions between brain regions should be examined (Wang et al., 2014) in order to improve the spatiotemporal network model currently embedded in BNA analysis. Modeling techniques to be tested will include structural equation modeling (SEM, Büchel and Friston, 1997; Tsubomi et al., 2009), dynamic causal modeling (Friston et al., 2003; Moran et al., 2013), Granger causality (Ding et al., 2006; Deshpande et al., 2010) and multivariate regression (Friston, 1994; for a review, see Craddock et al., 2015).

ERP tasks (with emphasis on target vs. non-target differentiation) are also used by brain-computer interface (BCI) technologies. BCI technologies have proven to be effective at using single trial classification algorithms to detect targets in variety of ERP tasks (e.g., spatial common pattern in P300 speller, Lotte et al., 2007). The goals and assumptions of BCI research are different than that of the clinical group ERP analysis. A BCI algorithm learns the single subject pattern based on its own single trials in order to help impaired patients or to improve the single subject target detection. Group ERP analysis aims to find patterns common to a group of subjects. Those patterns can provide insights regarding the clinical status of a subject who did not participate in the pattern learning stage. By characterizing a certain clinical population, one can diagnose a patient based on the results of controlled experiments. Differentiating target and non-target single trials recorded from the same subject presents different challenges than differentiating the ERPs of different clinical groups. In single trials, the signal-to-noise ratio (SNR) is low but the differences between two stimuli in the same subject are large, while in ERP analysis the SNR is larger but the variability between subjects is also high which in turn lowers the differentiation ability of a group common pattern due to the large variance.

The large variability between subjects and the good repeatability of the ERPs raises two models to assess the changes of the ERPs across different sessions with an individual subject. The first one is to generate a network that represents a group of normal subjects as a reference and follow the evolution of the single subject's conformity to that of the group, as done in this paper. The second model is to record a baseline session of the subject, against which each session can be tested for conformity. The advantage of the second model is the ability to trace single subject changes that cannot be seen using similarity measures compared to the normal population due to the group variability. On the other hand, the main challenge in using the single subject baseline model is to define a meaningful change from the changes emerging from irrelevant personal and environmental parameters. The BNA algorithm is useful in these two aspects as it allows similarity to be graded between any two sessions as well as between a session and a derived group-common template. The set of STEPs representing the single subject can be compared to another set of STEPs generated in another session or to a reference BNA model.

In this study, BNA analysis was evaluated using an oddball ERP paradigm. BNA analysis extracted the ERP components reported in the literature, and it demonstrated repeatability across visits and accurate classification of Target and Novel stimuli. Future studies should examine the utility of BNA analysis in the clinical setting by evaluating the classification of patients and healthy controls and of several disease subtypes, while measuring both the stability of the features and the accuracy of the classifications.

## Author contributions

YS developed the concept of STEP, implemented the integration to BNA and performed the analysis. AG, AR developed the concept of BNA and supervised the STEP algorithm and the integration.

## Funding

This study is part of ElMindA's Brain Disorders Management and Imaging Technology program, which is supported by the Office of the Chief Scientist (OCS), Israel.

### Conflict of interest statement

AR, YS, and AG are employees of ElMindA Ltd., Herzliya, Israel. They are inventors on several patents related to the methods described in this manuscript.
