# Supplementary material for: Brain Network Activation Analysis Utilizing Spatiotemporal Features for Event Related Potentials Classification
Source: Front Comput Neurosci. 2016 Dec 20;10:137. doi: 10.3389/fncom.2016.00137 (PMC5167752; doi:10.3389/fncom.2016.00137)
Supplement: Supplementary file 1 [file DataSheet1.DOCX]

Supplementary Material

**Addition of Spatiotemporal Features into the Brain Network Activation Analysis for Improved Data Classification**

Yaki Stern^*^, Amir Reches, Amir Geva

*** Correspondence:** Yaki Stern, yaki@elminda.com

# Supplementary Methods

## The clustering procedure

The goal of the clustering was to identify a set of group STEPs using clusters of individual subjects’ STEPs. Therefore, each group STEP represents a spatiotemporal event common to all individual subjects included in a specific cluster. In order to achieve this goal, all the STEPs of all subjects were entered into the clustering procedure. Three constraints were imposed on the clustering procedure: (1) temporal and spatial windows were imposed on each cluster. The temporal windows were of the following lengths: 200, 125, 77 and 56 ms, corresponding to each of the four frequency bands of δ, θ, α, and β, respectively. The temporal windows were determined as factor of the average frequency of each frequency band. The spatial window was set to be equivalent to the minimal distance between all possible pairs of non-neighboring electrodes in the 10-20 system of a 64-channel electrode array. Both window constraints were applied to each of the STEP’s peaks; (2) only one STEP per subject was allowed to participate within a cluster and the same STEP could not have appeared in two different clusters; (3) each cluster must have contained at least 70% of the subjects that participated in the clustering procedure.

Clustering comprised three stages (Fig. 1C): a) mapping of all optional clusters; b) choosing the best clusters; and c) generating a group STEP for each cluster. In stage (a), all possible clusters were extracted under the constraints specified above. Sub-clusters that were contained within clusters with more participants were ignored. In stage (b), a greedy procedure was implemented. The aim of this procedure was to choose the highest quality clusters from those mapped in (a). The quality measure of a cluster was defined as a composite score determined by a combination of the following factors: the number of participating subjects, the average Euclidian distance between all individual peaks and the average topographic similarity across all STEPs in the cluster. In the third stage (c), group STEPs were extracted. The group STEP was calculated as the average of individual peaks and their surroundings. The group peak was calculated by averaging the individual peaks included in the cluster in three dimensions: space, time and amplitude. As a pre-processing stage preceding group STEP extraction, ERP extrapolation was performed. Specifically, the original high resolution ERP of every subject that participated in the cluster was extrapolated onto a larger spatial plane surrounding the recording surface (the extrapolated distance was defined as the distance between two neighboring 10-20 system electrodes). Then, the extrapolated ERP was shifted so that the individual subject’s peak was aligned with the group peak. Averaging all of the aligned ERPs resulted in a new, high-resolution, averaged ERP. Next, group STEPs were extracted following the same procedure that was performed at the single subject level (see “ERP data segmentation” in the methods section). The final output of clustering at the group level was a set of group STEPs distributed along each of the dimensions of the ERP activity (Fig. 1C and D).

# Supplementary Figures and Tables

## Supplementary Figures


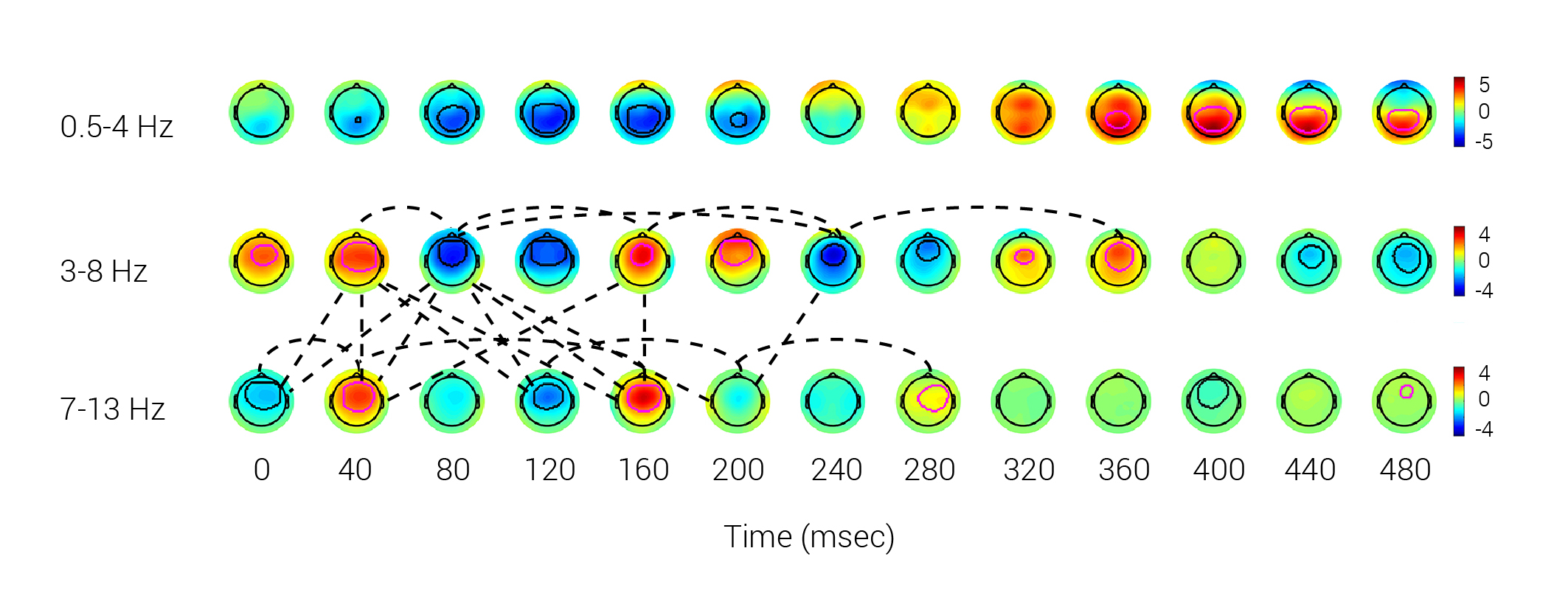
A


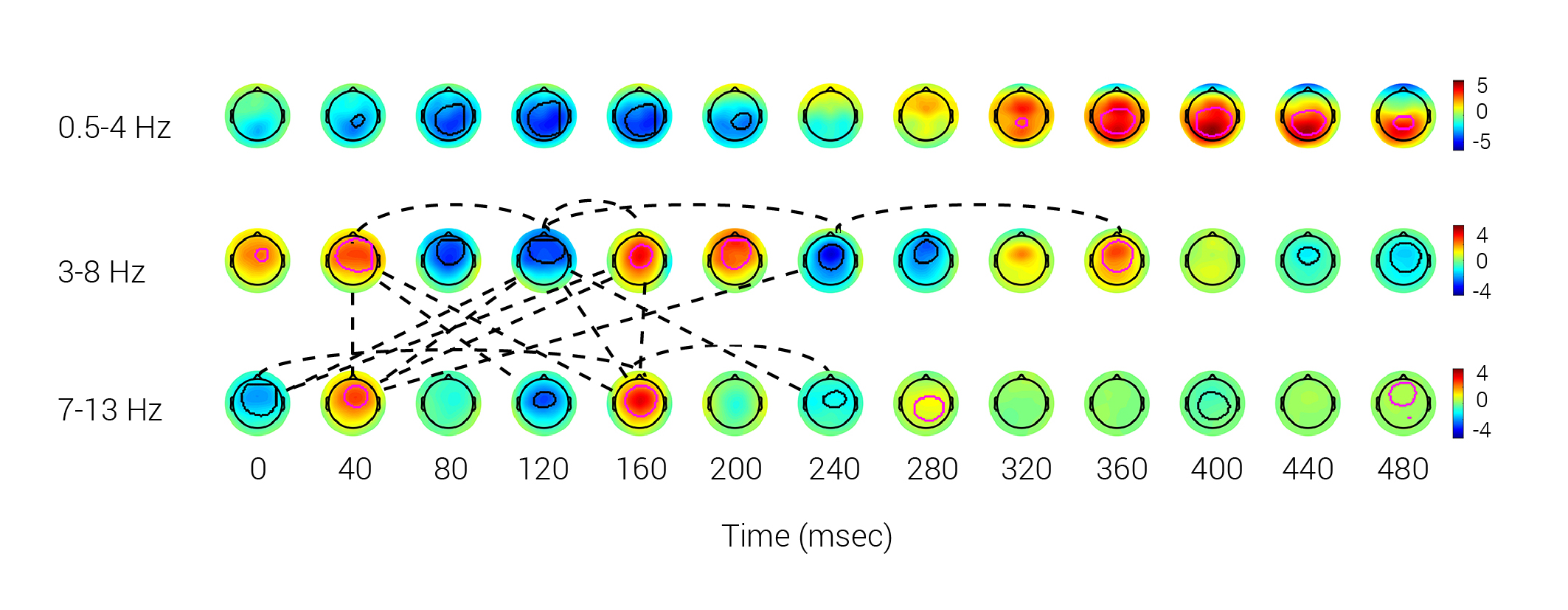
B

**Figure S1.** The Target group network for Group A: V1 (A) and V2 (B). The contours (thick lines) that appear inside the potential maps circumscribe each STEP’s peak and surroundings. A magenta contour represents a positive polarity group STEP, whereas a black contour represents a negative polarity group STEP. The dotted lines are the connections between the group STEPs. The repeatability of the main STEPs and connectivity structures across visits can be seen clearly.


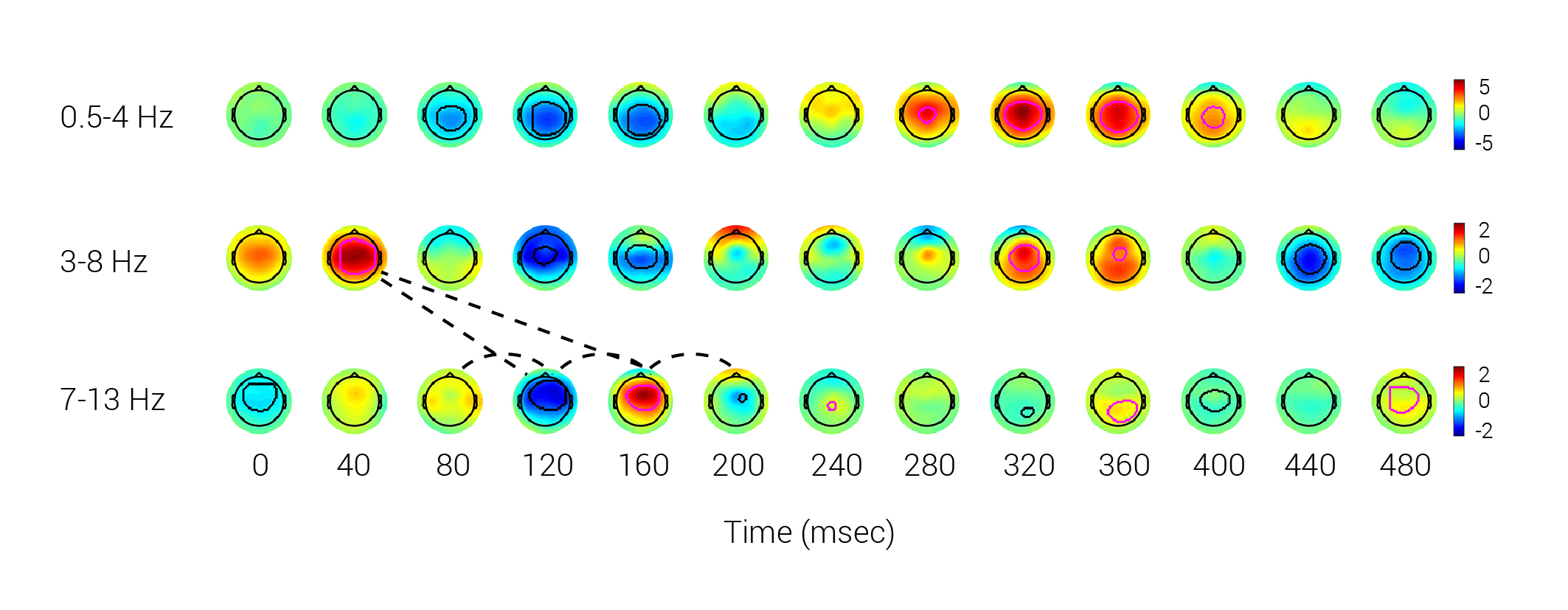


A


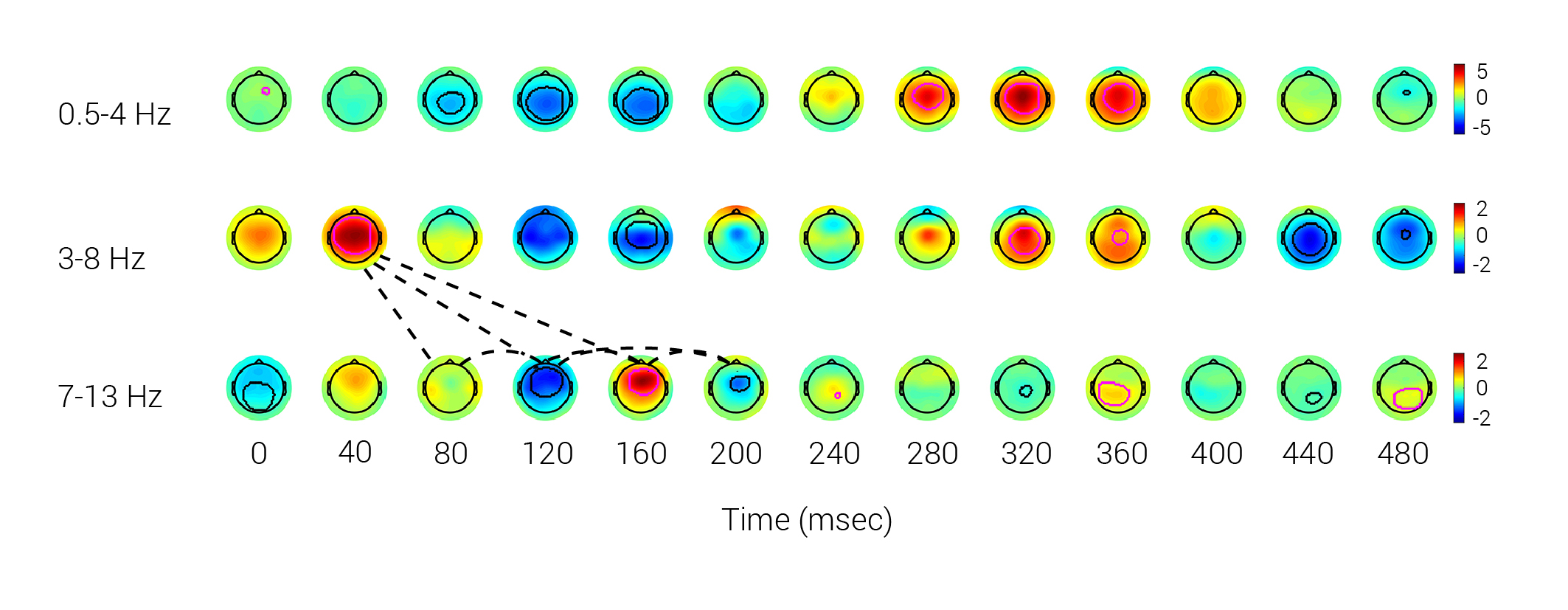


B

**Figure S2**. The Novel group network for Group A: V1 (A) and V2 (B). The contours (thick lines) that appear inside the potential maps circumscribe each STEP’s peak and surroundings. A magenta contour represents a positive polarity group STEP, whereas a black contour represents a negative polarity group STEP. The dotted lines are the connections between the group STEPs. The repeatability of the main STEPs and connectivity structures across visits can be seen clearly.
